# The CNS-penetrant soluble guanylate cyclase stimulator CYR119 attenuates markers of inflammation in the central nervous system

**DOI:** 10.1186/s12974-021-02275-z

**Published:** 2021-09-18

**Authors:** Susana S. Correia, Guang Liu, Sarah Jacobson, Sylvie G. Bernier, Jenny V. Tobin, Chad D. Schwartzkopf, Emily Atwater, Elisabeth Lonie, Sam Rivers, Andrew Carvalho, Peter Germano, Kim Tang, Rajesh R. Iyengar, Mark G. Currie, John R. Hadcock, Christopher J. Winrow, Juli E. Jones

**Affiliations:** 1 Cyclerion Therapeutics, 245 First St., Riverview II, 18th Floor, Cambridge, MA 02142 USA; 2 Ironwood Pharmaceuticals, Cambridge, MA 02142 USA

**Keywords:** Soluble guanylate cyclase, sGC, cGMP, Nitric oxide, CREB, Microglia, Neuroinflammation, Quinolinic acid, High-fat diet

## Abstract

**Background:**

Inflammation in the central nervous system (CNS) is observed in many neurological disorders. Nitric oxide-soluble guanylate cyclase-cyclic guanosine monophosphate (NO–sGC–cGMP) signaling plays an essential role in modulating neuroinflammation. CYR119 is a CNS-penetrant sGC stimulator that amplifies endogenous NO–sGC–cGMP signaling. We evaluated target engagement and the effects of CYR119 on markers of neuroinflammation in vitro in mouse microglial cells and in vivo in quinolinic acid (QA)-induced and high-fat diet-induced rodent neuroinflammation models.

**Methods:**

Target engagement was verified in human embryonic kidney (HEK) cells, rat primary neurons, mouse SIM-A9 cells, and in rats by measuring changes in cGMP and downstream targets of sGC signaling [phosphorylated vasodilator-stimulated phosphoprotein (pVASP), phosphorylated cAMP-response element binding (pCREB)]. In SIM-A9 cells stimulated with lipopolysaccharides (LPS), markers of inflammation were measured when cells were treated with or without CYR119. In rats, microinjections of QA and vehicle were administered into the right and left hemispheres of striatum, respectively, and then rats were dosed daily with either CYR119 (10 mg/kg) or vehicle for 7 days. The activation of microglia [ionized calcium binding adaptor molecule 1 (Iba1)] and astrocytes [glial fibrillary acidic protein (GFAP)] was measured by immunohistochemistry. Diet-induced obese (DIO) mice were treated daily with CYR119 (10 mg/kg) for 6 weeks, after which inflammatory genetic markers were analyzed in the prefrontal cortex.

**Results:**

In vitro, CYR119 synergized with exogenous NO to increase the production of cGMP in HEK cells and in primary rat neuronal cell cultures. In primary neurons, CYR119 stimulated sGC, resulting in accumulation of cGMP and phosphorylation of CREB, likely through the activation of protein kinase G (PKG). CYR119 attenuated LPS-induced elevation of interleukin 6 (IL-6) and tumor necrosis factor (TNF) in mouse microglial cells. Following oral dosing in rats, CYR119 crossed the blood–brain barrier (BBB) and stimulated an increase in cGMP levels in the cerebral spinal fluid (CSF). In addition, levels of proinflammatory markers associated with QA administration or high-fat diet feeding were lower in rodents treated with CYR119 than in those treated with vehicle.

**Conclusions:**

These data suggest that sGC stimulation could provide neuroprotective effects by attenuating inflammatory responses in nonclinical models of neuroinflammation.

## Background

Neurodegenerative diseases are complex and heterogenous, and yet chronic neuroinflammation is a core facet across diseases [1]. Soluble guanylate cyclase (sGC) is a signaling enzyme that is broadly expressed throughout the body, including in the central nervous system (CNS). sGC is activated by nitric oxide (NO), a critical neurotransmitter, and its activity results in the production of cyclic guanosine-3′,5′-monophosphate (cGMP) from guanosine-5′-triphosphate. Because the NO–sGC–cGMP signaling pathway modulates broad physiological mechanisms such as vasodilation, fibrosis, metabolism, and inflammation [2, 3], therapeutic targeting of this pathway may provide benefit in a wide range of diseases.

sGC stimulators are small molecules that potentiate the NO–sGC–cGMP signaling pathway by synergizing with endogenous NO. The anti-inflammatory effects of sGC stimulators have been broadly described in peripheral tissues. In models of systemic inflammation, sGC stimulators reduced levels of circulating inflammatory markers [4] and markers of vascular inflammation [5]. For instance, in the Dahl salt-sensitive rat model of hypertension, sGC stimulators decreased the levels of plasma interleukin 6 (IL-6) [4]. In other models of organ injury, sGC stimulators reduced markers of local inflammatory response in lung [6] and in liver [7, 8]. However, the effects of sGC stimulation on inflammation in the CNS have not been well characterized, in large part due to the lack of availability of a CNS-penetrant sGC stimulator. Recently, we described the first CNS-penetrant sGC stimulator in clinical development and its ability to improve neuronal activity, mediate neuroprotection, and increase cognitive performance in nonclinical models [9]. Although the impact of sGC stimulation on neuroinflammation remains to be determined, cGMP signaling has been implicated in inflammatory response modulation in the CNS. In microglial cultures, the cell-permeable cGMP analog, 8-bromoguanosine (8-Br)-cGMP, reduced activation of the nuclear factor kappa-B (NF-κB) pathway [10]. Inhibitors of phosphodiesterase 5 (PDE-5), an enzyme that degrades cGMP, increased cGMP in microglia cultures and suppressed inflammatory markers triggered by treatment of these cultures with lipopolysaccharides (LPS) [11]. In brain slice cultures, inhibitors of sGC or protein kinase G (PKG), a downstream target of cGMP, exacerbated dopaminergic cell death induced by interferon gamma/LPS, while 8-Br-cGMP reduced cytotoxicity [12]. These data suggest that modulation of the NO–sGC–cGMP pathway can impact neuroinflammation. The goal of this study was to explore the role of the sGC pathway in neuroinflammation using the CNS-penetrant sGC stimulator CYR119 in multiple in vitro and in vivo models.

## Methods

### Animal husbandry

Rats and mice were housed individually in polycarbonate cages with filter tops and were acclimated for at least 3 days before study start under controlled conditions of temperature (22 ± 4 °C) and relative humidity (30–70%), in a 12:12-h light–dark cycle room (lights on at 6:30 A.M.) at an AAALAC-accredited animal research facility. Unless noted, animals were allowed ad libitum access to water and standard rodent chow (Harlan Teklad, Indianapolis, IN; Irradiated Teklad Global 16%).

### Statistical analysis

Unless noted, statistical significance was determined by analysis of variance (ANOVA). A significant main effect was followed with an appropriate post hoc test. An effect was considered significant if *p* < 0.05. Data are presented as the mean ± standard error of the mean (SEM) and were graphed and analyzed using GraphPad Prism (v 9).

### Measurement of cGMP using the GloSensor cell-based assay

Human embryonic kidney 293 (HEK293) cells expressing the cGMP sensor, GloSensor™ 40F clone (Promega, Cat. #CS182801), were maintained in modified Eagle medium (DMEM) supplemented with fetal bovine serum (FBS) (10% final) and hygromycin B (200 µg/mL). For sGC activity assays, cells were seeded in DMEM with 10% FBS in a 50-µL volume at a density of 1.5 × 10^4^ cells/well in a poly-d-lysine coated, 384-well, white, flat-bottom plate (Corning, Cat. #35661). Cells were incubated overnight at 37 °C in a humidified chamber with 5% CO_2_. The next day, medium was replaced with 2 mM GloSensor reagent (Promega, Cat. #E1291) at 40 µL/well in CO_2_-independent medium (Gibco Cat. #18045-088). Cells were treated for 90 min (min) at 25 °C to allow the reagent to equilibrate in the cells. Diethylenetriamine NONOate (DETA) was added to the wells to make a concentration of DETA solution (0–30 µM) along with CYR119 at final concentrations of 0.029, 0.114, 0.460, 1.83, 7.32, 29.29, 117.2, 468.8, 1875, 7500, and 30,000 nM. After a 50-min incubation at room temperature, the luminescence signal generated by the interaction of intracellular cGMP with GloSensor 40F protein was measured with Envision Multilabel Reader (Perkin Elmer, Model #2104-0010A). Data were analyzed with a 4-parameter fit [log(agonist) vs. response − variable slope]. The EC_50_, defined as the concentration at which a given compound elicited 50% of the maximal response, was interpolated for CYR119 from the curve fit.

### Preparation of rat primary neurons culture

Neurons were isolated from Sprague Dawley rat embryos on embryonic Day 18. Hippocampus and cortex were dissected from the brains under a stereoscopic microscope. The tissues were dissociated and washed gently once with 10 mL of Ca^2+^ and Mg^2+^ free HBSS (Corning, Cat. #21-022-CM) in a 15-mL conical tube. After washing, 5 mL of a solution consisting of 0.25% trypsin (Invitrogen, Cat. #15090-046) and 0.1% deoxyribonuclease I (DNase I; Sigma, Cat. #DN-25) were added to the tissues and incubated at 37 °C for 15 min. Tissues were washed 3 times with ice-cold HBSS, and 3 mL of 0.1% of DNase I solution was added. Tissues were slowly pipetted 12 times using a glass Pasteur pipette followed by centrifugation at 500×*g* for 10 min. The resulting cell pellet was resuspended in culture medium (Neurobasal medium; Gibco, Cat. #21103-049), 2% of B27 supplement (Gibco, Cat. #17504-044), 0.5 mM l-glutamine (Corning, Cat. #25-005-Cl), 25 µM L-glutamic acid (Sigma, Cat. #G1251), and 1% penicillin/streptomycin (Gibco, Cat. #15070-063). The cell suspension was plated into poly-l-lysine-coated 384-well plates (for cGMP assay) or 96-well plates (for phosphorylated cAMP-response element binding [pCREB] assay) at 30,000 or 100,000 cells/well, respectively. Twenty-four hours after plating, half of the culture medium was removed and replaced with culture medium described above, without glutamic acid. Cells were maintained in a 37 °C humidified incubator with 5% CO_2_ and used on Days 6 through 10.

### cGMP measurement in rat primary neurons

Rat primary neurons were washed once with HBSS with calcium and magnesium and then incubated with a solution containing 0.5 mM 3-isobutyl-1-methylxanthine (IBMX) in HBSS (80 µL/well) at 37 °C for 15 min. A 5X stock (20 µL) of CYR119 with fixed concentration of DETA (30 µM) was added to the wells to make the desired concentration for CYR119 (0.003, 0.015, 0.077, 0.384, 1.92, 9.6, 48, 240, 1200, 6000, and 30,000 nM). Cells were incubated for 20 min at 37 °C and cGMP was measured in cell lysate by liquid chromatography with tandem mass spectrometry (LC–MS/MS). Briefly, a standard curve of the internal standard was prepared in 10% acetic acid (aqueous) containing 13C cGMP (Toronto Research Chemicals). Cell lysates and standards were analyzed for cGMP and internal standard levels using a Thermo Vantage Triple Quadrupole LC–MS in positive Ionization mode, linked to an Acquity ultra-performance liquid chromatography instrument (Waters Corporation, Milford, MA). Samples were loaded onto a Thermo Hypersil Gold 2.1 × 50-mm, 1.9-micron particle-size column. The mobile phase consisted of aqueous 0.1% formic acid (mobile phase A) and acetonitrile with 0.1% formic acid (mobile phase B). The flow rate was 0.750 mL/min. The gradient was held at 100% mobile phase A for 0.2 min, ramped to 50% mobile phase A by 0.3 min, and held for 0.7 min before returning to 100% mobile phase A by 0.8 min. Total run time was 1 min per sample. Data were fit using a 4-parameter fit [log(agonist) vs. response − variable slope]. The EC_50_ for CYR119 was interpolated from the curve fit.

### pCREB measurement in rat primary neurons

Rat primary neurons were washed once with HBSS with calcium and magnesium and then incubated with 90 μL HBSS for 30 min at 37 °C. A 10X stock (10 µL) of CYR119 with fixed concentration of DETA (10 µM) was added to the wells to make the desired concentration for CYR119 (0.01, 0.1, 1, 10, 100, 1000, and 10,000 nM). During development of the assay, the concentration of DETA (10 µM) was chosen because it had minimal activity on CREB phosphorylation alone and provided sufficient exogenous NO to enable CYR119 to elicit a response. Cells were incubated for an additional 30 min at 37 °C. Medium was removed, cells were lysed, and levels of pCREB and CREB were determined according to Cisbio protocols (phospho-CREB [Ser133], Cat. #64CREPEG and total CREB, Cat. #63ADK052PEG). Plates were read using an Envision instrument (PerkinElmer). pCREB/CREB ratios were determined and analyzed with a 4-parameter fit (log(agonist) vs. response −  variable slope). The EC_50_ for CYR119 was interpolated from the curve fit.

### pVASP stimulation in SIM-A9 cells

Mouse microglial SIM-A9 cells were purchased from ATCC (Cat. #CRL-3265) and cultured according to ATCC recommended growth conditions in DMEM-F12 (ATCC, Cat. #30-2006) containing 10% heat-inactivated fetal bovine serum (HI-FBS; Gibco, Cat. #16140-071) and 5% heat-inactivated horse serum (HI-HS; Gibco, Cat. #26050-088).

To determine phosphorylated vasodilator-stimulated phosphoprotein (pVASP) formation, SIM-A9 cells were seeded in 96-well V-bottom cell culture plate (Corning Costar #3894) at 100,000 cells/well/200 µL in complete culture medium (DMEM-F12 containing 10% HI-FBS and 5% HI-HS). The cell pellets were centrifuged (Beckman Model #TJ-25) at 1500 rpm for 5 min, then washed twice with HBSS solution containing Ca^2+^ and Mg^2+^ (Gibco Cat. #14025-075). Cells were pre-incubated with 90 µL/well of HBSS containing 0.5 mM IBMX at 37 °C for 15 min. After pre-incubation, 10 µL of CYR119 alone or in combination with DETA (diluted in HBSS/0.5 mM IBMX) were added to the cells and further incubated at 37 °C for 20 min. After incubation, supernatant was removed, and cells were lysed for pVASP and total VASP determination using CisBio homogenous time-resolved fluorescence (HTRF) assays (phospho-VASP [Ser239], Cat. #63ADK065PET and total VASP, Cat. #63ADK067PEG). To detect the formation of pVASP-Ser239 and total VASP in the cells, cell pellets were lysed with CisBio lysis buffer (Cat. #64KL2FDF) and then 16 µL cell lysates were transferred to a ProxiPlate 384-well plate (Perkin Elmer part #6008230) and assayed following the Cisbio pVASP HTRF assay kit protocol.

### Measurement of LPS-induced cytokines in SIM-A9

SIM-A9 cells were seeded in 96-well V-bottom cell culture plate at 200,000 cells/well/180 µL in plain DMEM-F12 medium and incubated at 37 °C for 5 h (cells were serum-starved during this period). At the end of this 5-h period, the cells were incubated with 10 µL of 20 × stocks of CYR119 diluted in DMEM-F12 medium at 37 °C for 30 min, followed by the addition of 10 µL of LPS at 20 × of its final concentration (100 ng/mL) and further incubation for 18 h at 37 °C. Cell supernatants were collected and levels of secreted IL-6 and TNF were determined using respective ELISA assay kits from R&D Systems (Cat. #M6000B and #MTA00B, respectively) following manufacturer’s protocols. Values were extrapolated from the standard curve, which resulted in vehicle-treated wells having negative values for protein level. Data were analyzed by one-way ANOVA followed by Bonferroni’s multiple comparisons test to LPS control wells.

### CSF collection from rats

Male Sprague Dawley rats implanted with an intracisternal cannula (250–275 g, Charles River) were used for this study. Rats were acclimated to the animal facility at Cyclerion Therapeutics for at least 3 days before study start and were fasted overnight before compound administration. On the experimental day, rats were orally (P.O.) dosed with vehicle (0.5% methyl cellulose [MC]/0.5% TWEEN^®^ 80) or CYR119 (10 mg/kg). Inclusion of an additional NO donor, such as DETA, was not necessary for in vivo experiments due to availability of endogenous NO. For CSF sampling, animals were anesthetized with isoflurane and the implanted cannula was cleared by withdrawing and discarding 20 µL of CSF. Immediately after, approximately 50 µL of CSF were withdrawn and collected into Eppendorf tubes containing 5 µL of glacial acetic acid and then snap-frozen in liquid nitrogen. CSF was collected 1, 2, and 4 h after dosing for quantification of CYR119 exposure. CSF was collected 1 h after dosing for quantification of cGMP.

### Measurements of CYR119 and cGMP in biological matrices

The method to quantify CYR119 and cGMP in rat plasma and CSF samples was developed at Cyclerion Therapeutics. For determination of CYR119 and cGMP levels, a standard curve was prepared in plasma and artificial CSF (with 20% acetic acid and 0.05% bovine serum albumin) by serial dilution. Acetonitrile containing an internal standard for each analyte was added to samples, which were then vortexed and centrifuged. Supernatants were transferred to a 96-well plate and dried under N_2_ gas. Dried samples were reconstituted with 0.1% formic acid in water. Samples were analyzed for CYR119 and cGMP levels using a Sciex 6500 QTrap coupled to an Agilent Technologies 1260 Infinity LC System. The effect of CYR119 on cGMP was analyzed by an unpaired two-tailed t-test.

### Quinolinic acid (QA) rat model

Male Sprague Dawley rats (300 g, Charles River) were used for this study. Rats were anesthetized and mounted in a stereotaxic frame and 0.25 µL of 50 mM quinolinic acid (QA) were unilaterally infused at 0.1 µL/min in the dorsal striatum (AP: + 1; ML: ± 2.5; DV: 4.2). A vehicle infusion of the same volume was performed in the contralateral dorsal striatum. Rats were randomly assigned to receive either vehicle (0.5% MC/0.5% Tween 80) or CYR119 (10 mg/kg) dosed daily for 7 days. The first dose was delivered subcutaneously approximately 30 min after the QA infusion, and then once daily P.O. for the next 6 days. Brain tissue was collected 24 h after the last P.O. dose. Rats were processed for immunohistochemistry (vehicle *n* = 5, CYR119 *n* = 6) or gene expression (vehicle *n* = 6, CYR119 *n* = 6), as described below.

### Immunohistochemistry

Rats were deeply anesthetized with isoflurane and transcardially perfused with 4% paraformaldehyde prepared in phosphate buffer saline (PBS) buffer. Whole brain was collected and incubated in 4% paraformaldehyde for 16 h, transferred to 30% sucrose prepared in PBS, and stored at 4 °C for at least 48 h before tissue processing. Each brain was sectioned in 40-µm-thick coronal slices. Sections containing striatum were stained with anti-Iba1 (Wako: 019-19741; diluted 1:500), anti-glial fibrillary acidic protein (GFAP; Cell Signaling Technology: #3670; diluted 1:300), or anti-pCREB (Millipore: 06-519; diluted 1:500) antibodies.

Sections were incubated with secondary antibody goat anti-rabbit conjugated to Alexa Fluor 594 (Iba1) and goat anti-mouse conjugated to Alexa 488 (GFAP and pCREB), both diluted 1:200 in blocking buffer (0.3% Goat serum, 0.3% Triton X-100, 0.2% Tween, 10% 10X PBS in water). Sections were imaged on a Zeiss fluorescence confocal microscope with a 25 × objective and 0.5 × Zoom for GFAP and pCREB images and 10 × objective and 0.6 × Zoom for Iba1 images and analyzed with ImageJ software. Quantitation of fluorescence was performed in the dorsomedial area around the striatal lesion and analyzed as percent area of antibody staining (GFAP and IBA1) or intensity (cumulative distribution and average intensity; pCREB).

### Diet-induced obese (DIO) mice

Six-week-old male C57BL/6 mice (Jackson Laboratories) were placed on 60% high-fat diet (HFD) for 10 weeks, which induced obesity (DIO), insulin resistance, and increased proinflammatory cytokines [13]. Starting at 16 weeks of age, animals received daily P.O. administration of vehicle (0.5%MC/0.5% Tween 80; *n* = 10) or CYR119 (10 mg/kg, *n* = 10) for 6 weeks and were maintained on HFD. Prior to killing, mice were fasted for 3 h and administered CYR119 or vehicle 1.5 h before killing. Prefrontal cortex was microdissected and rapidly frozen; mRNA levels were determined by QuantiGene multiplex assay. A chow-fed (*n* = 8; no dose) group was included as the lean control.

### Gene expression analyses

To analyze gene expression levels in rats and mice in the QA and DIO experimental paradigm, samples of rat striatum and mouse prefrontal cortex were homogenized and processed using a QuantiGene sample processing kit (Affymetrix Cat. #QS013) in accordance with manufacturer’s instructions. Gene expression in the tissue was measured using a QuantiGene 2.0 Plex Assay kit (Affymetrix Cat. #QP1013) and a multiplex gene panel (Thermo/Fisher, plex set #331368 for rat; plex set #QGP-150-M18033001 for mouse) according to manufacturer guidelines. Analytes were measured using Luminex MAGPIX™ (Bio-Rad, Hercules, CA). Median fluorescence intensity was generated for each gene target and normalized to the geometric mean of housekeeping genes [ribosomal protein L19 (Rpl 19) and actin beta for rat; peptidylprolyl isomerase B, Rpl19, and RNA polymerase II subunit A for mouse], which were chosen to match the target transcript abundance.

## Results

### In vitro activity of CYR119

**Fig. 1:**
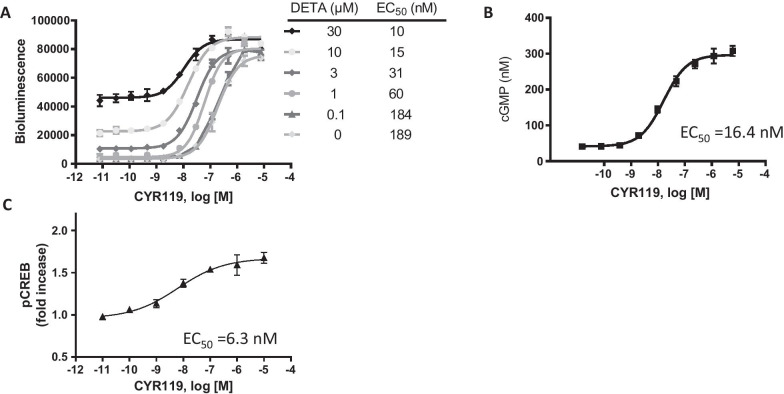
In vitro activity of CYR119. **A** GloSensor^®^ luciferase assay in HEK293 cells in the presence of 0 to 30 µM DETA-NONOate. **B**, **C** Effect of CYR119 on cGMP and CREB phosphorylation in rat primary neurons. Representative concentration response of CYR119 as measured by cellular phosphorylation of CREB in the presence of 10 µM DETA-NONOate in rat primary neurons. Data are normalized to vehicle-control wells. EC_50_s are noted in each figure

In the cell-based cGMP GloSensor® luciferase assay, CYR119 increased intracellular cGMP in the absence of exogenously applied NO (EC_50_ = 189 nM); application of DETA (an NO donor at 0.1 to 30 µM) increased the measured potency of CYR119 to an EC_50_ of 10 nM, demonstrating the synergistic behavior of CYR119 with NO, which is typical of sGC stimulators [2] (Fig. 1A). In rat primary neurons, CYR119 induced production of cGMP (EC_50_ = 16.4 nM) and stimulated the phosphorylation of CREB (EC_50_ = 6.3 nM) in a concentration-dependent manner (Fig. 1B, C).

**Fig. 2:**
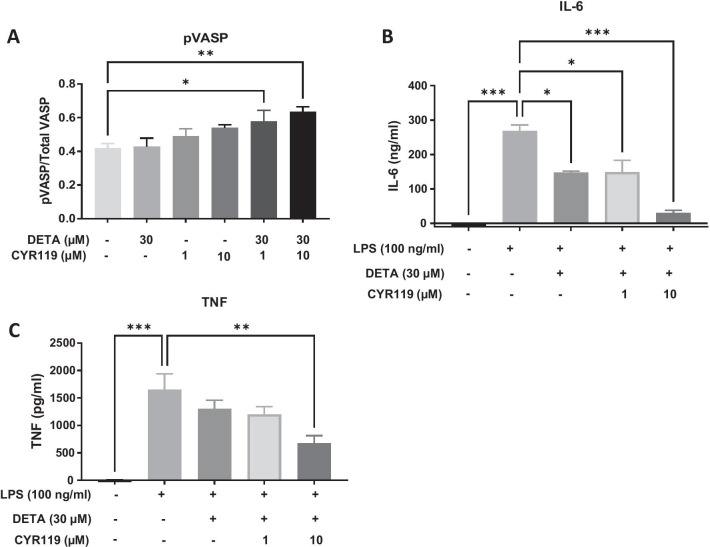
Measurement of LPS-induced cytokines in SIM-A9 following incubation with CYR119. **A** Phosphorylation of VASP (pVASP/total VASP) in SIM-A9 cells after incubation with CYR119 (1 or 10 µM) with or without DETA (30 µM). Data analyzed with a one-way ANOVA followed by a Dunnett’s multiple comparison test vs. vehicle-control wells. **B**, **C** Effect of CYR119 ± 30 µM DETA on IL-6 and TNF protein production, respectively, in SIM-A9 cells after LPS stimulation. Data analyzed with a one-way ANOVA followed by a Bonferroni’s multiple comparison test vs. LPS-treated control wells. Data are expressed as mean ± SEM; *p < 0.05, **p < 0.01, ***p < 0.001, non-significant (ns) effects are not highlighted

Phosphorylation of VASP, a PKG target, at Ser239 [14] was measured after treating SIM-A9 cells with CYR119 alone or in combination with DETA. Stimulation with CYR119 at 1 or 10 µM in combination with 30 µM DETA increased phosphorylation of VASP (Fig. 2A), confirming the presence of the sGC-cGMP signaling pathway in these cells.

To investigate the effect of CYR119 on LPS-induced cytokine production, SIM-A9 cells were treated with 100 ng/mL of LPS for 24 h, which resulted in increased levels of IL-6 and TNF protein. The LPS-mediated increase in IL-6 expression was attenuated in cells incubated with 1 or 10 µM CYR119 and 30 µM DETA (Fig. 2B), while LPS-mediated TNF expression was lower in cells incubated with 10 µM CYR119 and 30 µM DETA compared with LPS vehicle-treated cells (Fig. 2C).

### In vivo CNS exposure of CYR119

**Fig. 3:**
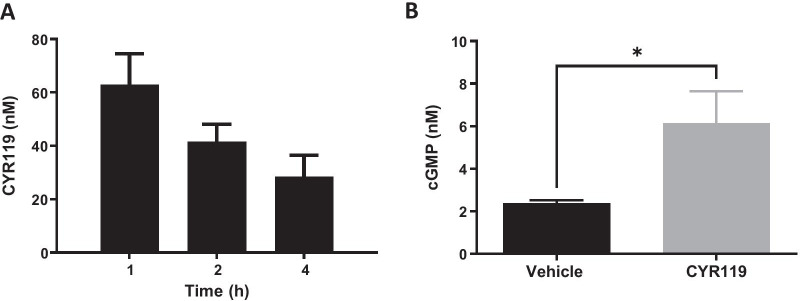
Concentration of CYR119 and cGMP in rat CSF following oral delivery. **A** Hour post-dose compound concentration (nM) in the CSF of rats administered 10 mg/kg CYR119. **B** One hour after dosing, concentrations of cGMP in the CSF were higher in rats dosed orally with 10 mg/kg CYR119 vs. vehicle-treated rats (*p < 0.05; two-tailed *t*-test). Data are expressed as mean ± SEM

The ability of CYR119 to cross the blood–brain barrier (BBB) and stimulate cGMP production in the CNS was determined in rats with a cisterna magna cannulation after P.O. administration of CYR119 (10 mg/kg) or vehicle. CYR119 was detected in the CSF at 1, 2, and 4 h after dosing, indicating brain penetrance (Fig. 3A). Furthermore, cGMP levels were greater in rat CSF samples collected 1 h after oral dosing with CYR119 (10 mg/kg) than in samples collected from vehicle-treated rats (Fig. 3B), further supporting CNS target engagement.

### CYR119 suppressed QA-induced neuroinflammatory markers and increased pCREB in rat brain

**Fig. 4:**
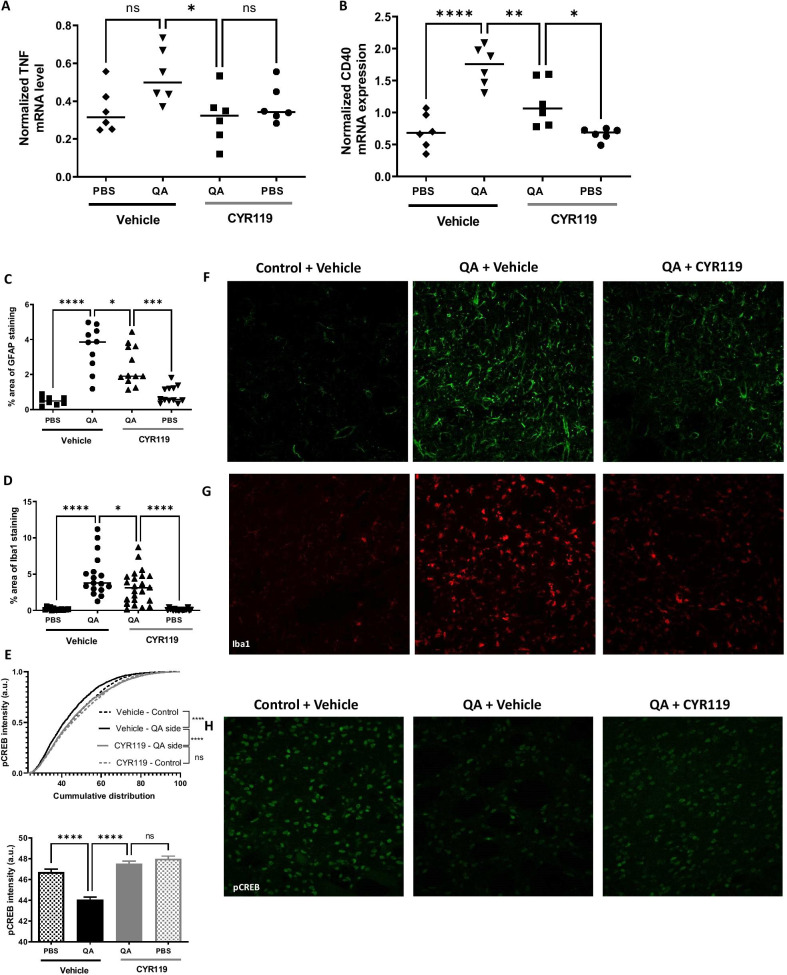
Effects of CYR119 on markers of inflammation following QA administration. Rats were administered 10 mg/kg CYR119 or vehicle for 7 days following QA administration into the dorsal striatum. Normalized gene expression was analyzed for **A** TNF and **B** CD40 or immunohistochemistry staining of **C** glial fibrillary acidic protein (GFAP), and **D** IBA1, represented as percent (%) area of Iba1 staining, and **E** pCREB, represented as intensity as a cumulative distribution and average intensity in the dorsal striatum. A representative image from each group is shown in **F** GFAP, **G** IBA1, and **H** pCREB. Data were analyzed by one-way ANOVA followed by Sidak’s multiple comparison test (*p < 0.05, **p < 0.01, ***p < 0.001, ****p < 0.0001). Data are expressed as mean ± SEM

Since we demonstrated that CYR119 can cross the BBB in vivo and can reduce neuroinflammatory markers in vitro, we next hypothesized that CYR119 would reduce neuroinflammation in vivo. To test this hypothesis, a model of QA-induced neurodegeneration that is characterized by an increase in local neuroinflammation in the brain was used [15]. All rats received a single unilateral infusion of 12.5 nmoles of QA in the dorsal striatum and a single infusion of PBS in the contralateral dorsal striatum, and were then treated with either vehicle or CYR119 (10 mg/kg) once per day for 7 days. As expected, QA infusion induced an increase of TNF and CD40 mRNA in the dorsal striatum of vehicle-treated rats as compared with the contralateral dorsal striatum (PBS-injected control side; Fig. 4A, B). Additionally, QA infusion resulted in greater GFAP (astrocyte marker) and Iba1 (microglia marker) protein levels as measured by immunostaining around the QA infusion site vs. the same brain area on the contralateral control side (Fig. 4C, D). These data indicate that QA infusion induced a local neuroinflammatory response around the infusion site. The TNF and CD40 mRNA levels in the QA-injected dorsal striatum of rats treated with CYR119 were lower than in vehicle-treated rats (Fig. 4A, B). In agreement with these results, the staining for GFAP and Iba1 protein levels around the QA infusion site of rats treated with CYR119 was lower than in rats that were treated with vehicle (Fig. 4F, G). In summary, these data indicate that oral administration of CYR119 reduced local brain inflammatory response in the QA rat model.

To further understand the effects of CYR119 in the CNS, we explored the modulation of phospho-CREB (pCREB), a downstream target of NO–sGC–cGMP pathway [16]. pCREB is a critical signaling molecule in the CNS that can reduce neuroinflammation by increasing expression of anti-inflammatory cytokines [17]. In primary hippocampal neurons, treatment with CYR119 (10 pM–10 µM) for 30 min increased pCREB (Fig. 1C). As we observed reduction of neuroinflammatory markers after treatment with CYR119 in the QA model, we further examined the effect of CYR119 on pCREB levels in this model. In vehicle-treated rats, pCREB immunostaining was less pronounced in the dorsal striatum injected with QA than in the contralateral control side (Fig. 4E and H). Average intensity of pCREB immunostaining around the QA lesion was greater in animals treated with CYR119 than in vehicle-treated rats (Fig. 4E and H). These data demonstrate that CYR119 increased pCREB in the same brain area where markers of neuroinflammation were lower.

### Expression of inflammatory genes was attenuated in the cortex of obese mice treated with CYR119

**Fig. 5:**
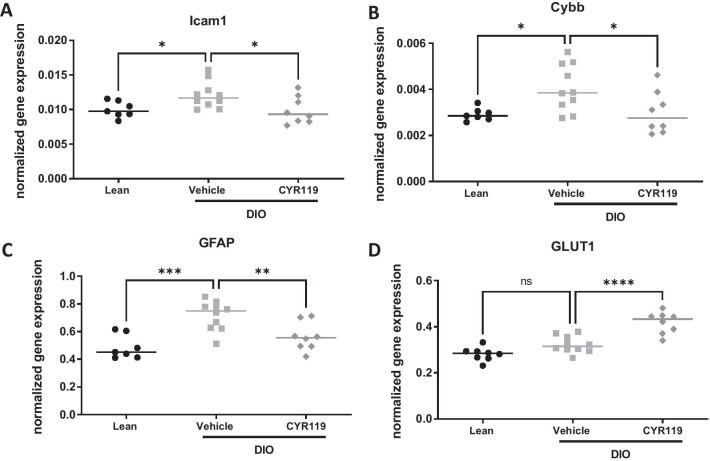
Effects of CYR119 on inflammatory genes in the prefrontal cortex of DIO mice. CYR119 (10 mg/kg) was orally administered to DIO mice for 6 weeks and prefrontal cortex analyzed for gene expression of **A** intercellular adhesion molecule 1 (ICAM1), **B** NADPH oxidase 2 (Cybb), **C** glial fibrillary acidic protein (GFAP), and **D** glucose transporter 1 (GLUT1). Data were analyzed with a one-way ANOVA followed by a Dunnett’s multiple comparison test vs. DIO vehicle-control mice. Data are expressed as mean ± SEM; *p < 0.05, **p < 0.01, ***p < 0.001, ****p < 0.0001

In addition to obesity, consumption of a high-fat diet can lead to neuroinflammation, which is marked by changes in the expression of proinflammatory cytokines [13]. In this study, DIO mice were treated with either vehicle or CYR119 for 6 weeks. Body weight was similar in CYR119- and vehicle-treated DIO mice throughout the study (data not shown). A standard (lower fat) chow-fed group was included as a lean control. In the prefrontal cortex, gene expression levels of intercellular adhesion molecule 1 (ICAM1), NADPH oxidase 2 (Cybb), and GFAP were higher in DIO mice than in lean mice, while levels of ICAM1, Cybb, and GFAP gene expression were lower in DIO mice treated with CYR119 for 6 weeks than in DIO vehicle-treated mice (Fig. 5). Expression of glucose transporter 1 (GLUT1) was higher in DIO mice treated with CYR119 than in DIO vehicle-treated or chow-fed mice. Endothelin 1 gene expression was greater in DIO vehicle-treated mice than lean mice but remained unchanged in CYR119-treated mice (data not shown). The inflammatory genes, Vasp, nuclear factor NF-kappa-B, chemokine (C–C motif) ligand (Ccl) 3, Ccl11, mitogen-activated protein kinase 8 (MAPK8), toll-like receptor 4 (TLR4), and vascular cell adhesion protein 1 (VCAM1), were similar between all three groups (data not shown). Expression levels of IL-6, IL-10, IL-1β, Ccl2, Ccl11, and TNF were below detection and thus were not analyzed.

## Discussion

NO, a ubiquitous signaling molecule and neurotransmitter, modulates various biological processes and is associated with both pro- and anti-inflammatory effects in the periphery and the CNS [2, 18]. NO binds to and activates sGC to generate cGMP. While many of the downstream effects of NO are mediated by sGC, the functions of NO and sGC do not completely overlap because NO can signal through other pathways, including through protein nitrosylation and the generation of nitro-oxidative stress. Therefore, sGC stimulators are a more selective way to amplify cGMP signaling compared to NO donors [19, 20]. Pharmacotherapies aimed at increasing the level of cGMP have focused both upstream (NO donors, sGC stimulators, and sGC activators) and downstream (PDE inhibitors) of cGMP [2, 21]. PDEs are a class of enzymes that degrade cyclic nucleotides such as cGMP. PDE inhibitors have been shown to modulate inflammation by preserving existing levels of cGMP [11, 22]. Because sGC stimulators increase the production of cGMP rather than preventing its degradation, they may have a more robust effect on increasing cGMP levels and have the potential for greater anti-neuroinflammatory effects in diseases associated with NO deficiency. Therefore, we explored the anti-inflammatory effects of increasing cGMP via sGC stimulation by using the CNS-penetrant sGC stimulator CYR119 in nonclinical models of neuroinflammation. We demonstrated that CYR119 is an orally bioavailable sGC stimulator that can enhance the activity of the NO–sGC–cGMP pathway in the CNS. In vitro, CYR119 increased the production of cGMP in HEK cells and showed synergistic effects with the NO donor DETA. Additionally, in primary neurons, CYR119 together with DETA elicited increases in cGMP levels. Activation of cGMP downstream signaling was also observed in microglial cells where sGC stimulation by CYR119 increased VASP phosphorylation. In rats, CYR119 increased cGMP levels in CSF after oral dosing, consistent with results from preclinical studies of the clinical-stage CNS-penetrant sGC stimulator CY6463, which also demonstrated an increased in CSF cGMP. The ability of CNS-penetrant sGC stimulators to increase cGMP levels in the CSF is in contrast to that of CNS-restricted sGC stimulators that do not alter CSF cGMP levels [9]. Consistent with previous work showing that increased NO–sGC signaling can modulate inflammation [18, 23], we found that CYR119 reduced the LPS-induced inflammatory response in cultured microglial cells, reduced QA-induced neuroinflammation in rats [15], and reduced inflammatory gene expression in a DIO mouse model that is an established model of neuroinflammation [24].

In response to a CNS insult such as a QA injection, an inflammatory response is often initiated by activation of toll-like receptors (TLR) in astrocytes, which in turn increases expression of GFAP and leads to astrogliosis. TLRs signal via MyD88 to activate both MAPK and IκB, which in turn trigger the phosphorylation of AP-1 and nuclear translocation of NFκB, respectively. In the nucleus, pancreatitis-associated protein I (pAP-1) and NFκB promote a transcriptional cascade that releases proinflammatory cytokines including IL-6, IL-1β, TNF, and IL-12 [25]. Our work demonstrates that GFAP and Iba1 expressions around the site of QA injury were lower in animals treated with CYR119 than with vehicle, indicating that CYR119 reduces activation of astrocytes and microglia in this model. Additionally, in the DIO mouse model, GFAP expression in the prefrontal cortex, which is one of the regions in the brain linked to cognitive decline observed in obesity [26], was lower in DIO mice treated with CYR119 than in the DIO-control mice. The reduced astroglial activation is consistent with previous studies showing that the NO–sGC–cGMP pathway modulates neuroinflammation through activation of PKG, which inhibits LPS-induced expression of MMP-9, a process that is dependent on NFκB activation [27]. In human endothelial cells, NO suppressed NADPH oxidase-dependent superoxide production [28]. NADPH oxidase-2 (Cybb) plays a role in the inflammatory response by producing reactive-oxygen-species in response to an insult. Inhibitors of this pathway are being developed to potentially treat inflammation [29]. In our study, Cybb gene expression in the prefrontal cortex was greater in vehicle-treated mice fed an HFD than in obese animals treated with CYR119. This effect of the NO–sGC–cGMP pathway has been proposed to contribute to the resolution of inflammation [30]. During CNS injury, microglia known to play a critical role in neuroinflammation produce IL-1β, TNF, IL-6, and other cytokines as the astrocytes undergo morphological changes. In our research, CYR119 reduced gliosis at the site of QA injury, which may explain the reduction of proinflammatory cytokine transcription (e.g., TNF) that was observed in the brain of QA rats. In fact, in LPS-treated microglial cultures, levels of IL-6 and TNF were lower in the culture media of cells incubated with CYR119 that with vehicle, indicating that the sGC stimulator can reduce secretion of these proinflammatory markers from microglial cells. Collectively, these results suggest that the ability of CYR119 to lower CNS inflammation may be partially mediated by the resolution of gliosis.

One potential component of the inflammatory response is the increase of trans-endothelial transport of immune cells across the BBB. This increase is in part mediated by the increase of ICAM-1 and VCAM-1 in the endothelial cells surrounding the BBB vasculature and results in increased adhesion of leukocytes, lymphocytes, monocytes, eosinophils, and basophils to the vasculature, ultimately facilitating the infiltration of these cells through the BBB. In the DIO mouse model, we observed a higher expression of ICAM-1 mRNA in the prefrontal cortex of obese mice than in lean control mice. However, ICAM-1 mRNA expression in the prefrontal cortex was lower in obese mice treated with CYR119 than in obese control mice, suggesting that CYR119 may reduce the BBB dysfunction triggered by inflammation. These results are consistent with previous work showing that in a mouse model of cerebral malaria, treatment with an NO-donor decreased expression of ICAM-1 and P-selectin in the brain [31]. Future work with CYR119 or another CNS-penetrant sGC stimulator such as CY6463 [9] in additional models with more pronounced cytokine expression will increase the understanding of the role of the NO–sGC–cGMP pathway in neuroinflammation.

In an in vitro model of polymorphonuclear leukocyte (PMN) adhesion to human brain microvessel endothelial cells (HBMEC), both NO donors and 8-Br-cGMP decreased adhesion of PMN to HBMEC induced by TNF [32, 33]. Previous work also revealed that a peripherally restricted sGC stimulator prevented the increase of intravascular inflammation as reflected by attenuated plasma levels of soluble platelet-selectin, soluble endothelial-selectin, and soluble intercellular adhesion molecule-1 (sICAM-1) in a model of TNF-induced inflammation [34]. Additionally, an sGC stimulator has been shown to suppress leukocyte-endothelial cell interactions [34]. These findings are consistent with data showing that nitric oxide synthase regulates leukocyte–endothelial cell interactions [35, 36].

In addition to reducing inflammation, while CYR119 did not elicit any alterations in body weight, GLUT1 expression was higher in the prefrontal cortex of DIO animals treated with CYR119 than in vehicle-treated mice. GLUT1 is responsible for glucose transport across the BBB and is critical for BBB development [37, 38]. Activation of the NO–sGC–cGMP pathway has been shown to increase glucose uptake via GLUT1 [39]. Reduction of glucose transport into the brain because of lower GLUT1 expression has been described in human diseases that have CNS inflammation such as Alzheimer’s disease and other neurodegenerative diseases [37], and GLUT1 deficiency in the endothelium is involved in the breakdown of the BBB [24]. In our study, although high-fat feeding did not lead to a decrease in GLUT1 expression in the prefrontal cortex, GLUT1 expression was increased in CYR119-treated animals, possibly reflecting enhanced glucose transport into the brain, which may counteract the dysfunction elicited by HFD. In addition, hypothalamic brain-derived neurotrophic factor (BDNF) expression was higher in DIO mice treated with the CNS-penetrant sGC stimulator CY6463 than in DIO-control mice [9], and restoration of GLUT1 has been shown to increase BDNF [40]. Since in the DIO mouse model the inflammatory insult involves a peripheral component, further work comparing peripheral versus CNS‑penetrant sGC stimulators is essential for dissecting the multiple pathways by which sGC stimulation may regulate neuroinflammation.

## Conclusions

The data presented here from preclinical models demonstrate that the CNS-penetrant sGC stimulator CYR119 can increase cGMP levels and reduce neuroinflammation in the CNS. The data highlight the potential of CNS-penetrant sGC stimulators to treat neurological disorders that have an inflammatory component.

## Data Availability

The datasets used and/or analyzed during the current study are available from the corresponding author upon reasonable request.

## Abbreviations

ANOVA: Analysis of variance
BBB: Blood–brain barrier
BDNF: Brain-derived neurotrophic factor
cGMP: Cyclic guanosine monophosphate
CNS: Central nervous system
CSF: Cerebrospinal fluid
Cybb: NADPH oxidase 2
DETA: Diethylenetriamine NONOate
DIO: Diet-induced obesity
DNase I: Deoxyribonuclease I
EC_50_: Concentration at which a compound elicits the half-maximal response
FBS: Fetal bovine serum
GFAP: Glial fibrillary acidic protein
GLUT1: Glucose transporter 1, also known as SLC2A1
HBMEC: Human brain microvessel endothelial cells
HEK: Human embryonic kidney
HFD: High-fat diet
HI: Heat inactivated
HS: Horse serum
HTRF: Homogenous time-resolved fluorescence
Iba1: Ionized calcium binding adaptor molecule 1
IBMX: 3-Isobutyl-1-methylxanthine
ICAM1: Intercellular adhesion molecule 1, also known as CD54
IL-6: Interleukin 6
LC–MS/MS: Liquid chromatography with tandem mass spectrometry
LPS: Lipopolysaccharide(s)
MAPK8: Mitogen-activated protein kinase 8
MC: Methyl cellulose
min: Minute(s)
NF-κB: The nuclear factor kappa-B
NO: Nitric oxide
PBS: Phosphate buffer saline
pCREB: Phosphorylated cAMP-response element binding
PDE: Phosphodiesterase
PKG: Protein kinase G
PMN: Polymorphonuclear leukocyte
P.O.: Oral(ly)
pVASP: Phosphorylated vasodilator-stimulated phosphoprotein
QA: Quinolinic acid
Rpl 19: Ribosomal protein L19
sICAM-1: Soluble intercellular adhesion molecule-1
SEM: Standard error of the mean
sGC: Soluble guanylate cyclase
TLR: Toll-like receptor
TNF: Tumor necrosis factor
VCAM 1: Vascular cell adhesion protein 1
vs.: Versus
